# Changes of arterial pressure following relief of obstruction in adults with hydronephrosis

**DOI:** 10.1080/03009734.2018.1521890

**Published:** 2018-10-08

**Authors:** Ammar Al-Mashhadi, Michael Häggman, Göran Läckgren, Sam Ladjevardi, Tryggve Nevéus, Arne Stenberg, A. Erik G. Persson, Mattias Carlström

**Affiliations:** a Pediatric Surgery Section, Department of Women’s and Children’s Health, Uppsala University, Uppsala, Sweden;; b Department of Surgical Sciences, Uppsala University, Uppsala, Sweden;; c Pediatric Nephrology Unit, Department of Women’s and Children’s Health, Uppsala University, Uppsala, Sweden;; d Department Medical Cell Biology, Uppsala University, Uppsala, Sweden;; e Department of Physiology and Pharmacology, Karolinska Institutet, Stockholm, Sweden

**Keywords:** Blood pressure, hydronephrosis, hypertension, kidney, renal function, ureteral obstruction

## Abstract

**Background:** As much as 20% of all cases of hypertension are associated with kidney malfunctions. We have previously demonstrated in animals and in pediatric patients that hydronephrosis causes hypertension, which was attenuated by surgical relief of the ureteropelvic junction (UPJ) obstruction. This retrospective cohort study aimed to investigate: (1) the proposed link between hydronephrosis, due to UPJ obstruction, and elevated arterial pressure in adults; and (2) if elevated blood pressure in patients with hydronephrosis might be another indication for surgery.

**Materials and methods:** Medical records of 212 patients undergoing surgical management of hydronephrosis, due to UPJ obstruction, between 2000 and 2016 were assessed. After excluding patients with confounding conditions and treatments, paired arterial pressures (i.e. before/after surgery) were compared in 49 patients (35 years old; 95% CI 29–39). Split renal function was evaluated by using mercaptoacetyltriglycine (MAG3) renography before surgical management of the hydronephrotic kidney.

**Results:** Systolic (−11 mmHg; 95% CI 6–15 mmHg), diastolic (−8 mmHg; 95% CI 4–11 mmHg), and mean arterial (-9 mmHg; 95% CI 6–12) pressures were significantly reduced after relief of the obstruction (*p* < 0.001). Split renal function of the hydronephrotic kidney was 39% (95% CI 37–41). No correlations were found between MAG3 and blood pressure level before surgery or between MAG3 and the reduction of blood pressure after surgical management of the UPJ obstruction.

**Conclusions:** In adults with hydronephrosis, blood pressure was reduced following relief of the obstruction. Our findings suggest that elevated arterial pressure should be taken into account as an indication to surgically correct hydronephrosis.

## Introduction

Cardiovascular disease, including hypertension (≥140/90 mmHg) is a major health problem, associated with increased morbidity and mortality. Secondary forms of hypertension are found in 5–20% of the hypertensive population, and most of these cases can be linked to renal abnormalities (1,2), but the role of hydronephrosis has so far received only scant attention. Published reports of hypertension obviously caused by hydronephrosis are few, and the numbers of patients included in these reports are low (3,4).

The treatment of symptomatic ureteropelvic junction (UPJ) obstruction is surgical. It has been shown that the function of the hydronephrotic kidney is rather well preserved in young children (5–7). Based on this finding, the management policy concerning asymptomatic hydronephrosis has consequently become much more conservative. Besides the need for lifelong follow-up with repeated investigations, the potential cardiovascular risk of this new treatment strategy is still unknown.

Previous experimental studies in animals have demonstrated that hydronephrosis, due to chronic partial unilateral ureteral obstruction, is linked to the development of renal injuries and hypertension (8–14). Moreover, the causal relationship is demonstrated by the fact that arterial pressure is significantly lowered by surgical relief of the obstruction (15). In children, there are currently only a few reports investigating the association between hydronephrosis and abnormal arterial pressure (16,17). In a recent prospective study, we demonstrated that arterial pressure and markers of oxidative stress were elevated in pediatric patients with hydronephrosis compared with healthy controls, and that these disturbances were normalized following surgical management of the obstruction. The aim of this study was to investigate the hypothesis that arterial pressure preoperatively is higher than arterial pressure postoperatively in patients aged more than 15 years old. If correct, elevated blood pressure in patients with hydronephrosis should be considered as another indication for surgery.

## Material and methods

We studied the medical records and hospital charts of 212 patients with hydronephrosis, due to congenital UPJ, who were operated between 2000 and 2016 at the Urology Department of Uppsala University Hospital (Table 1). All patients were more than 15 years old.

**Table 1.** Characteristics of the population with UPJ obstruction.

Total population	*n* = 212
Age (y)	37 (95% CI 35–40)
Female/male (%)	53/47
Unilateral/bilateral (%)	93/7
Chronic disorders (%)	20
CVD, including hypertension (%)	15
Others (%)	12
Antihypertensive drugs (%)	10
Pre relief of UPJ obstruction (*n*)	*n* = 203
Systolic pressure (mmHg)	133 (95% CI 130–136)
Diastolic pressure (mmHg)	81 (95% CI 79–83)
Mean arterial pressure (mmHg)	99 (95% CI 97–100)
Post relief of UPJ obstruction (*n*)	*n* = 60
Systolic pressure (mmHg)	122 (95% CI 119–127)*
Diastolic pressure (mmHg)	76 (95% CI 72–78)*
Mean arterial pressure (mmHg)	91 (95% CI 88–94)*

* *p* < 0.005 compared with pre relief of UPJ obstruction.

CVD: cardiovascular disease; UPJ: ureteropelvic junction.

**Figure 1.**
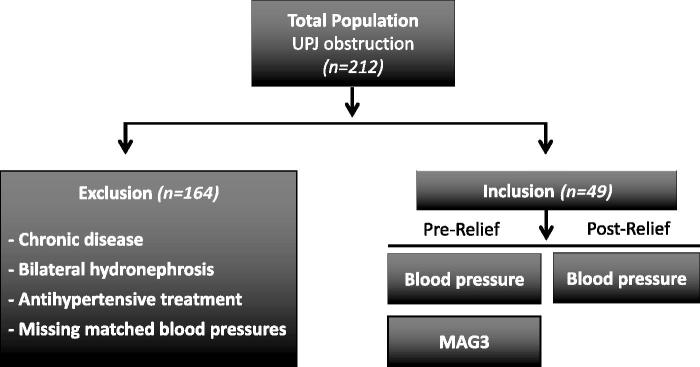
Schematic illustration of the study diagram. Medical records and hospital charts of 218 patients who were operated (2000–2016 at the Urology Department of Uppsala University Hospital) due to hydronephrosis were studied. A total of 212 patients displayed hydronephrosis due to ureteropelvic junction (UPJ) obstruction. Patients with other chronic disorders, bilateral hydronephrosis, antihypertensive treatment, or with missing data on blood pressure (i.e. before or after surgical management) were not included in the analysis. From the total population with operated UPJ obstruction 49 patients fulfilled the inclusion criteria, and their systolic, diastolic, and mean arterial pressure were analyzed before and after surgery, and linear regression analysis was carried out comparing changes in arterial pressure with renal function by mercaptoacetyltriglycine (MAG3) renography.

From the total population with operated UPJ, 49 patients fulfilled our inclusion criteria, i.e. patients with unilateral hydronephrosis not associated with any other chronic diseases that may be predisposing for hypertension (e.g. cardiovascular disease, diabetes, obesity), who were not treated with antihypertensive drugs, and with available arterial pressure values before and after surgical management of the hydronephrosis (Figure 1, Table 2). According to medical records the majority of UPJ was associated with aberrant vessel (*n* = 37; 76%), and to a lesser extent it was due to inflammation and fibrosis (*n* = 8; 16%) and pelviureteral junction stricture (*n* = 4; 8%). We included those blood pressure measurements when the patients were not in an acute situation nor in pain for any reason, even before temporary treatment with nephropyelostomy catheter or double J-stent. The same method of blood pressure monitoring (i.e. office) was used for all patients, and it was consistent in terms of location and time of the day (i.e. daytime). The procedure for all included patients was office blood pressure measurements during non-stressed situations and in the absence of any acute pain. We performed previously a similar study in which we included two control groups, i.e. one healthy not operated group and one healthy operated group and found no effects of preoperative stress on the blood pressure in patients with hydronephrosis (18). Moreover, due to the retrospective nature of this study, and that blood pressure results were acquired from the patient’s digital record, it is difficult to know if the measurements were made in supine or prone position, but the procedure should be the same for each patient.

**Table 2.** Patient characteristics pre- and postoperatively.

Age	Sex	Operation	Side	MAG3	AP (Pre)	AP (Post)
16	F	Lap pyeloplasty	Rt	34%	138/78	120/80
16	M	Lap pyeloplasty	Lt	55%	115/75	120/70
17	F	Lap pyeloplasty	Rt	50%	105/65	115/65
17	M	Robotic pyeloplasty	Lt	46%	130/80	120/80
17	M	Lap pyeloplasty	Lt	Not done	130/80	130/70
19	F	Lap pyeloplasty	Lt	20%	120/90	120/81
19	M	Lap pyeloplasty	Lt	47%	130/70	130/65
19	M	Lap pyeloplasty	Rt	30%	120/90	120/60
20^a^	F	Robotic pyeloplasty	Lt	54%	100/60	95/55
20	M	Lap pyeloplasty	Lt	41%	110/70	110/70
20	M	Lap pyeloplasty	Lt	41%	110/70	110/70
21	F	Lap pyeloplasty	Lt	37%	135/80	110/60
21	F	Lap pyeloplasty	Rt	41%	110/70	120/70
22	F	Lap pyeloplasty	Rt	30%	120/80	110/60
22	M	Lap pyeloplasty	Rt	23%	123/81	120/85
22	M	Lap pyeloplasty	Lt	37%	160/85	120/85
23^a^	M	Robotic pyeloplasty	Rt	40%	140/80	105/63
24	M	Lap pyeloplasty	Rt	47%	145/90	120/80
25	F	Lap pyeloplasty	Lt	41%	120/80	100/80
25	M	Lap pyeloplasty	Lt	48%	130/75	120/80
26	F	Robotic pyeloplasty	Lt	38%	120/75	120/70
26	M	Lap pyeloplasty	Lt	19%	170/105	130/90
26	M	Lap pyeloplasty	Lt	Not done	140/90	120/70
27	M	Lap pyeloplasty	Rt	Not done	110/80	108/73
32	F	Lap pyeloplasty	Rt	Not done	105/60	110/63
32	F	Robotic pyeloplasty	Rt	28%	165/110	138/90
33	F	Lap pyeloplasty	Rt	25%	110/65	104/70
35	F	Lap pyeloplasty	Rt	39%	120/80	100/70
35	M	Lap pyeloplasty	Lt	Not done	154/109	140/95
36	F	Lap pyeloplasty	Lt	39%	120/80	110/80
37	F	Lap pyeloplasty	Lt	60%	120/75	120/75
37	M	Robotic pyeloplasty	Rt	15%	130/90	120/70
41	F	Open pyeloplasty	Rt	31%	150/90	120/80
41	F	Lap pyeloplasty	Lt	62%	125/100	125/90
41	M	Lap pyeloplasty	Lt	48%	160/80	136/84
43^a^	M	Lap pyeloplasty	Rt	Not done	145/80	150/95
44	F	Lap pyeloplasty	Rt	46%	130/80	140/90
46^a^	M	Lap pyeloplasty	Rt	Not done	145/90	120/70
49	M	Lap pyeloplasty	Rt	38%	130/82	130/85
50	F	Robotic pyeloplasty	Rt	29%	130/80	120/85
50^a^	F	Robotic pyeloplasty	Rt	42%	150/100	120/70
50	M	Robotic pyeloplasty	Rt	40%	130/95	130/65
55	M	Lap pyeloplasty	Rt	Not done	160/110	140/95
56	F	Lap pyeloplasty	Rt	39%	140/90	140/90
67	F	Lap pyeloplasty	Rt	57%	169/117	140/90
68	F	Lap pyeloplasty	Lt	31%	111/82	110/60
68	F	Lap pyeloplasty	Rt	58%	195/90	150/90
71^a^	F	Lap pyeloplasty	Lt	39%	190/70	150/80
71	M	Lap pyeloplasty	Lt	Not done	160/90	150/70

a Patients who had recurrent ureteropelvic junction obstruction after pyeloplasty.

AP: arterial pressure (mmHg); F: female; Lap: Laparoscopic; Lt: left kidney; M: male; MAG3: mercaptoacetyltriglycine; Rt: right kidney.

Twenty-five (52%) of these patients had right side hydronephrosis. Thirty-eight patients were operated by laparoscopic pyeloplasty, while nine patients were operated by robot-assisted pyeloplasty, and one patient underwent open surgery. The patients’ age at the time of surgery ranged from 16 to 71 years, and 24 (49%) of them were males.

Renal obstruction was relieved temporarily before pyeloplasty with a double J-stent and/or percutaneous nephropyelostomy in 40 patients. The duration of this preoperative relief of obstruction ranged between two weeks and one year. Nephrostomy was performed preoperatively due to acute pain during the period the patient was waiting for the pyeloplastic surgery. Pre-relief arterial pressure was measured one day to one year before temporary relief of obstruction in these 40 patients, while pre-relief arterial pressure for the rest of patients was measured one day before pyeloplastic surgery. Post-relief arterial pressures were measured between two weeks to two years after relief of obstruction.

Seven (14.3%) of the investigated 49 patients had recurrent UPJ obstruction after pyeloplasty, but in the total population the recurrence frequency was 6.6% (14 of 212 patients). Three of these had undergone robotic pyeloplasty, while four relapses occurred after laparoscopic pyeloplasty (Table 3). Four of these seven patients were operated again with open pyeloplasty, whereas two were nephrectomized and one underwent ureterotomy. The arterial pressure of these seven patients was measured again before and after relief of the obstruction relapse.

**Table 3.** Recurrent cases of UPJ obstruction with pre-relief (before second operation) and post-relief (after second operation) arterial pressure, and methods of relief of the obstruction for the second time.

Age	Sex	Operation	Side	AP (Pre)	AP (Post)	Delta MAP
20	F	Pyeloplasty	Lt	110/60	95/50	12
23	M	Pyeloplasty	Rt	118/67	105/63	7
41	M	Nephrectomy	Lt	160/80	136/84	6
43	M	Ureterotomy	Rt	150/95	140/90	4
46	M	Pyeloplasty	Rt	140/80	120/75	10
50	F	Pyeloplasty	Rt	175/90	Missed	–
71	F	Nephrectomy	Lt	140/80	Missed	–

AP: arterial pressure (mmHg); F: female; Lt: left kidney; M: male; MAP: mean arterial pressure (mmHg); Rt: right kidney.

As a part of the routine management, preoperative evaluations of bilateral renal function were carried out using mercaptoacetyltriglycine (MAG3) renography with forced diuresis. This examination was performed in a standardized fashion. In nine patients the preoperative MAG3 renography was substituted with computed tomography (CT) urography, which has been proven an accurate and useful non-invasive imaging technique for the surgical planning (treatment) of UPJ obstruction (19).

### Statistical analysis

Non-parametric Kruskal–Wallis test, followed by Dunn’s test, was used for multiple comparisons among groups. Matched blood pressure values (before and after relief) were analyzed by non-parametric Wilcoxon test (i.e. matched-pairs signed rank test). Linear regression analysis (least squares ordinary fit) was used to compare age-grouped populations (i.e. total population, ≤30 and >30 years of age). Computed *p* values are indicated, and *p* < 0.05 denotes statistical significance.

## Results

### Arterial pressure

**Figure 2.**
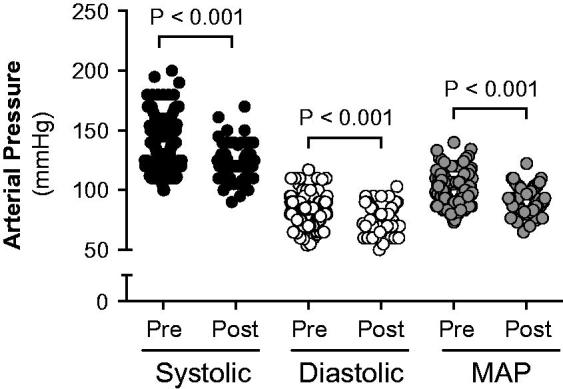
Systolic, diastolic, and mean arterial pressure (MAP) in hydronephrotic patients (*n* = 203; Male 47%; Female 53%) before (*n* = 203) and after (*n* = 60) surgical management of ureteropelvic junction obstruction.

**Figure 3.**
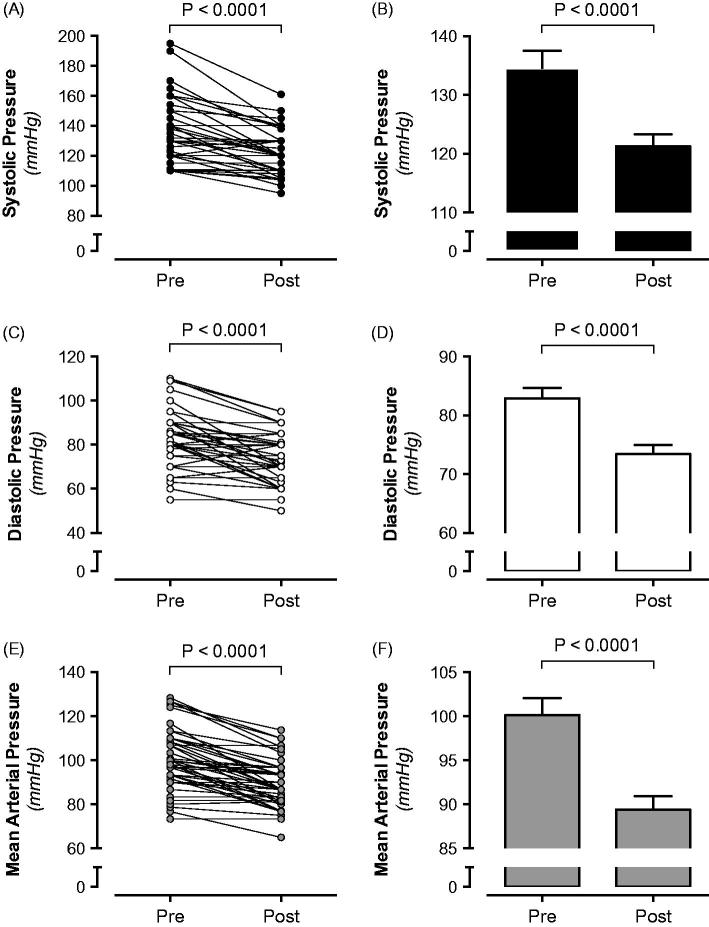
Matched systolic (A, B), diastolic (C, D), and mean arterial pressure (E, F) in hydronephrotic patients (*n* = 49; Male 49%; Female 51%) before and after surgical management of ureteropelvic junction obstruction.

In the total population of hydronephrotic patients, unpaired analysis suggested that systolic, diastolic, and mean arterial pressures (MAP) were higher before surgical management of UPJ (Table 1, Figure 2). After excluding patients with chronic disease, bilateral hydronephrosis, patients already on antihypertensive treatment, and those with missing blood pressure data, paired analysis of blood pressure values (i.e. pre and post relief) was performed on the remaining 49 patients. In agreement with the total population, systolic (Δ: −14 mmHg), diastolic (Δ: −10 mmHg), and MAP (Δ: −11 mmHg) were all significantly reduced after relief of the obstruction (*p* values <0.001 for all variables) (Table 2, Figure 3).

As mentioned above, seven patients had recurrence of UPJ stenosis 2–4 months after surgical management of the hydronephrosis and were thus re-operated (Table 3). Analysis of paired blood pressure values (*n* = 5), pre and post relief, showed significant reduction of systolic (Δ: −16 mmHg; *p* = 0.002) and MAP (Δ: −8 mmHg; *p* = 0.002), whereas diastolic pressure was not significantly reduced (Δ: −4 mmHg; *p* = 0.15).

**Figure 4.**
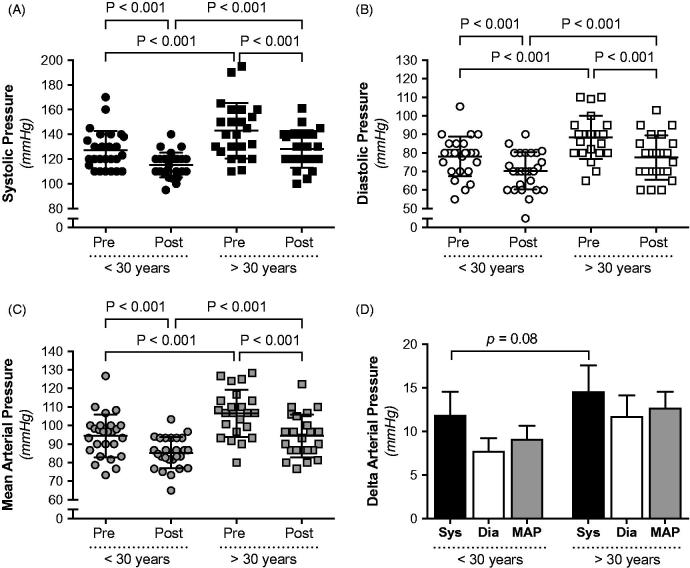
Systolic (A), diastolic (B), and mean arterial pressure (C) and changes in arterial pressure (D) in hydronephrotic patients separated in age groups (i.e. aged ≤30 years, or aged >30 years) before and after surgical management of ureteropelvic junction obstruction.

In order to investigate if the arterial pressure elevation and the reduction of blood pressure following surgery were influenced by age, a subanalysis was performed. The study population was divided into patients aged ≤30 years and patients aged >30 years at the time of surgery. Systolic, diastolic, and MAP were all significantly higher in the older patient group (Figure 4(A–C)). The blood pressure was significantly reduced following surgery in both age groups, and the magnitude of blood pressure was not significantly different, although there was a clear trend towards greater reduction in systolic arterial pressure in older patients (*P* = 0.08) (Figure 4(D)).

**Figure 5.**
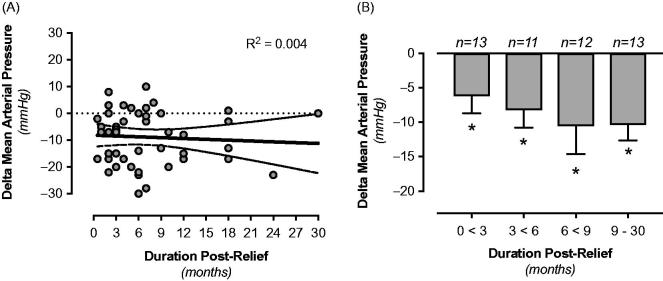
Impact of the length of the follow-up period before reassessing blood pressure after surgery. Linear regression analysis did not show any significant correlation between the length of the postoperative period and the change in arterial pressure (A). Significant reduction of blood pressure was observed in all groups compared with pre-relief values, independent of the length of the duration of the post-relief period, i.e. 0 to <3, 3 to <6, 6 to <9, and 9–30 months (B). No differences were observed among the different groups. *p < 0.05 compared with pre-relief; *n* = number of patients in each group.

Finally, a long follow-up period before reassessing blood pressure after surgery may influence the interpretation of the surgical intervention. In our study the shortest duration before obtaining post-relief values was 0.5 month, and the longest duration was 30 months. We stratified the blood pressure data based on the duration of the follow-up period. Linear regression analysis did not show any significant correlation between the length of the postoperative period and the change in arterial pressure (Figure 5(A)). When separating data into four groups with different time spans for the post-relief follow-up (i.e. 0 to <3, 3 to <6, 6 to <9, and 9–30 months), a significant reduction of blood pressure was observed in all groups compared with pre-relief values. However, there were no differences among the groups (Figure 5(B)).

### Renal function

Linear regression analysis did not reveal any correlation between systolic, diastolic, or MAP before surgery and the split renal function of the hydronephrotic kidney (MAG3) (Supplementary Figure S1(A–C), available online). In addition, there was no correlation between the changes in systolic, diastolic, or MAP following surgery and MAG3 (Supplementary Figure S2(A–C), available online). Further analysis, following separation by age groups (≤30 and >30 years of age) at the time of surgical management did also not reveal any significant correlations between reduction of blood pressure and MAG3 (Supplementary Figure S2(D–I), available online).

## Discussion

Unilateral hydronephrosis appears to be sufficient to cause elevation of blood pressure, regardless of the presence of a normal contralateral kidney (3). Some authors describe resolution of hypertension following removal of the affected kidney, while others—in agreement with our study—show that relief of obstruction may also lead to normalization of blood pressure. It therefore appears that the intrarenal mechanism leading to hypertension is reversible (3).

There are only few previously published case reports on the reduction or normalization of blood pressure following relief of renal obstruction in adults with hydronephrosis (3,20). Greminger et al. demonstrated that hypertension, associated with hydronephrosis, could be considered as a curable form of hypertension (21). In a case report by Chalisey et al., a patient was described presenting with acute kidney injury and bilateral hydronephrosis secondary to pelvic malignancy. Peripheral venous renin and aldosterone levels were elevated, and the patient was hypertensive. The high blood pressure reduced rapidly and was normalized 10 days following insertion of bilateral nephrostomies (22).

So far, only the study by Wanner et al. (4) addressed the question using a larger population. The authors found hypertension in 20% of 101 consecutive patients with unilateral hydronephrosis. These patients were followed for 35 months after surgery. Hypertension was cured in 62% of all cases, improved in 19%, and left unchanged in only 19% of the subjects. The authors concluded that, in unilateral hydronephrosis, high blood pressure is reversible by surgery (4).

In children with hydronephrosis with the same underlying pathology (i.e. UPJ obstruction), there are currently only three clinical studies that have investigated the effects of surgical relief of the obstruction on arterial pressure. The first study by de Waard et al. was a retrospective investigation that suggested reduction of arterial pressure after surgical management of dilated or obstructed upper urinary tracts (17). The second and third studies (16), conducted by our research group, were prospective and demonstrated that elevated systolic and diastolic pressures before surgery were significantly reduced following surgical correction of hydronephrosis in children with UPJ obstruction (16).

In the current study, we scrutinized all hydronephrotic patients (*n* = 212) that were operated in Uppsala University Hospital between 2000 and 2016, but in the final analysis only those patients with unilateral hydronephrosis due to UPJ obstruction that were not previously on antihypertensive therapy and had no underlying chronic disease were included. The exclusion of patients already on medication was made since the relationship (if any) between hydronephrosis and hypertension in such patients is difficult to evaluate. Moreover, it is very difficult to evaluate the short-term effect of obstruction relief on blood pressure if the patients are already on continuous antihypertensive treatment. We find it unlikely that this exclusion biases the results in a way that invalidates the conclusions made in this study. Of note, none of the patients included in this study initiated antihypertensive treatment after surgery or developed any other diseases before post-relief values were obtained. Life style questionnaires do not exist so we cannot exclude changes in their dietary habits or physical activity. However, no changes in BMI were observed between the pre- and post-relief examination.

Moreover, the retrospective nature of this study is associated with several limitations, e.g. our hospital is a tertiary center and patients come from other governorates’ hospitals. The evaluation and operation were performed at our clinical site, but the long-term follow-up of the patients was carried out in the governorates’ hospitals. Our hospital digital patient record system is limited, and we cannot access all patients’ follow-up visits in the governorates’ hospital patient record system. This limitation explains why repeated blood pressure and MAG3 data were not routinely obtained pre- and postoperatively for all the patients. The post-relief period in our study was variable, ranging from 0.5 to 30 months. Long duration before reassessment of blood pressure may certainly impact on the interpretation of the surgical intervention, since changes in many other parameters may occur. However, only few patients in this study had a post-relief period of more than 12 months. We found a similar degree of blood pressure reduction in all groups when data had been divided into different follow-up duration periods, thus supporting the conclusion that our results can be linked with the surgical intervention performed.

The current results are in agreement with previously mentioned clinical studies and case reports and also with our experimental research on hydronephrosis. We confirmed previously experimental (8,9,15) and clinical studies (16) that hydronephrosis is associated with elevated arterial pressure, which can be reduced by surgical relief. The current study also indicates that recurrence of obstruction, following surgical management of the hydronephrosis, was associated with elevated arterial pressure, and that the pressure was again reduced after relief of the obstruction. This finding gives further support to our hypothesis about the causal link between UPJ obstruction and elevation of the arterial pressure.

Findings demonstrating that the function of the hydronephrotic kidney is rather well preserved in young children (5–7) have led in recent decades to a shift towards non-surgical management of unilateral UPJ obstruction. A recent systematic review by Weitz et al. of non-surgical management of hydronephrosis could not resolve the ongoing controversy, and the authors concluded that recommendations cannot be made in favor of or against the non-surgical treatment of UPJ obstruction in children (23). As pointed out by the authors, in the short term approximately 80% of children with UPJ obstruction may be treated safely (at least regarding split renal function) with non-surgical management, but the follow-up periods available were too short to evaluate the long-term consequences. Against this background, the evidence for effective non-surgical management in children with unilateral UPJ obstruction needs to be critically assessed, accompanied by serial functional imaging with radiation exposure, anesthesia and/or analgosedation, which could potentially lead to serious adverse effects.

In a study by Menon et al. (24) in children with hydronephrosis and less than 20% renal function in the affected unit only three out of 62 patients developed hypertension, which in two cases resolved after surgery. Furthermore, they showed that no case of hypertension occurred in this group of patients without signs of obstruction after surgery, during the long-term follow-up (up to 8 years). In our current study, we tried to investigate if there is a correlation between renal function results according to MAG3 and blood pressure changes after relief of the obstruction. Based on findings in our current study, it was clear that in spite of preserved split renal function of the hydronephrotic kidney, the arterial pressure was elevated. Moreover, there was no correlation between MAG3 split renal function and the reduction in arterial pressure after relief of the obstruction. It has, however, previously been established that children with reflux nephropathy, i.e. renal parenchymal defects associated with vesicoureteral reflux, have an increased risk for hypertension (25). What is not known is whether this risk pertains to all scintigraphic uptake defects or only those that are due to acquired renal damage (‘scarring’) as opposed to congenital hypoplasias—since these subgroups are difficult or impossible to differentiate clinically. The same ambiguities pertain to children with hydronephrosis.

The current retrospective study does not provide evidence regarding the mechanism, but in several previous experimental studies we have investigated underlying mechanisms contributing to hydronephrosis-induced hypertension. These include increased adenosine receptor-mediated contraction in the kidney, activation of renin-angiotensin-aldosterone system, enhanced renal sympathetic nerve activity (8,11,13), together with altered prostaglandin and thromboxane signaling (26,27), oxidative stress, and impaired nitric oxide signaling in the affected kidney (10–13). In support of these findings, our recent clinical study also demonstrated that children with hydronephrosis have abnormal prostaglandin and thromboxane signaling, oxidative stress, nitric oxide homeostasis (18), and increased plasmin levels in the urine (28).

In conclusion, this study demonstrates that hydronephrosis in adult patients is associated with arterial pressure that is higher before relief of the renal obstruction if compared with values after relief of the obstruction. Importantly, higher arterial pressure, or the reduction of arterial pressure following surgery, did not significantly correlate with split renal function of the hydronephrotic kidney. Although future prospective studies using gold standard 24-h ambulatory blood pressure measurements with validated devices are warranted (29,30), we propose that arterial pressure measurements should be done routinely and frequently in patients with dilated or obstructed upper urinary tracts. Any sign of high arterial pressure (i.e. equal to or above 140/90 mmHg) in the absence of other chronic disorders should indicate the need for surgery. There are health benefits even with reduction of blood pressure from high normal to population median values. Our study suggests, but does not prove, that the urologist and nephrologist may have to pay more attention to the risk of development of high blood pressure in patients with hydronephrosis. The clinical importance of the current finding is that surgical management may prevent the development of chronic hypertension and associated comorbidities in patients with hydronephrosis.

## Ethical approval

All procedures performed in studies involving human participants were in accordance with the ethical standards of the institutional and/or national research committee (EPN; Protocol Number 2017/017, Uppsala, Sweden), and with the 1964 Helsinki declaration and its later amendments or comparable ethical standards.

## Supplementary Material

Supplemental Material
