# Supplementary material for: Changes of arterial pressure following relief of obstruction in adults with hydronephrosis
Source: Ups J Med Sci. 2018 Oct 8;123(4):216–24. doi: 10.1080/03009734.2018.1521890 (PMC6327611; doi:10.1080/03009734.2018.1521890)
Supplement: Supplemental Material [file IUPS_A_1521890_SM0768.docx]

**Changes of Arterial Pressure Following Relief of Obstruction in Patients with Hydronephrosis**

Ammar AL-MASHHADI^a^, Michael HÄGGMAN^b^, Göran LÄCKGREN^a^, Sam LADJEVARDI^b^, Tryggve NEVÉUS^c^, Arne STENBERG^a^, A. Erik G. PERSSON^d^, Mattias CARLSTRÖM^e^ *

^a^ Pediatric Surgery Section, Department of Women’s and Children’s Health, Uppsala University, Uppsala, Sweden.

^b^ Department of Surgical Sciences, Uppsala University, Uppsala, Sweden.

^c^ Pediatric Nephrology Unit, Department of Women’s and Children’s Health, Uppsala University, Uppsala, Sweden.

^d^ Department Medical Cell Biology, Uppsala University, Uppsala, Sweden.

^e^ Department of Physiology and Pharmacology, Karolinska Institutet, Stockholm, Sweden.

* Corresponding author

**Acknowledgements:** This work was supported by grants from the Swedish Research Council (2016-01381 MC, 65X-03522-43-3 AEGP), the Swedish Heart and Lung Foundation (20140448 MC, 20170124 MC), by Research Funds from the Karolinska Institutet (2-560/2015 MC) and Her Highness The Crown Princess Lovisa’s Fund for Scientific Research.

**Conflicts of interest:** NONE

**Correspondence:**

**Mattias Carlstrom**, Dept. of Physiology and Pharmacology, Karolinska Institutet, Nanna Svartz Väg 2, S-17177, Stockholm, Sweden.

Phone: +46-852487924.

Fax: +46-8332278

E-mail: [Mattias.Carlstrom@ki.se](mailto:Mattias.Carlstrom@ki.se)

**Figure S1.**

Linear regression analysis comparing systolic (A), diastolic (B) and mean arterial pressure (MAP) (C) with the renal function assessed by mercaptoacetyltriglycine (MAG3) renography in the total population of hydronephrotic patients. Data are presented with 95% confidence band of the best-fit line. R^2^, R square (*goodness of fit*).

**Figure S2.**

Linear regression analysis comparing changes in systolic, diastolic and mean arterial pressure (MAP) with the renal function assessed by mercaptoacetyltriglycine (MAG3) renography in age-grouped hydronephrotic patients, *i.e.,* total population (A-C), ≤ 30 (D-F) and > 30 years of age (G-I). Data are presented with 95% confidence band of the best-fit line. R^2^, R square (*goodness of fit*).
